# Ectopic Expression of *SjCBL1*, Calcineurin B-Like 1 Gene From *Sedirea japonica*, Rescues the Salt and Osmotic Stress Hypersensitivity in Arabidopsis *cbl1* Mutant

**DOI:** 10.3389/fpls.2018.01188

**Published:** 2018-08-21

**Authors:** Joo Hyuk Cho, Mi Na Choi, Kwan Hee Yoon, Kyung-Nam Kim

**Affiliations:** Department of Molecular Biology, PERI, Sejong University, Seoul, South Korea

**Keywords:** *Sedirea japonica*, calcium sensor, CBL, CIPK, salt stress, osmotic stress

## Abstract

Extensive studies with *Arabidopsis thaliana* suggested that calcineurin B-like (CBL) proteins constitute a unique family of calcium sensors in plants, which mediate a variety of abiotic stress responses. However, little is known about their function in most plants that do not have available genome sequences. In this study, we have developed a pair of universal primers that make it possible to isolate CBL1-like genes from various plants without sequence information. Using these primers, we successfully cloned a full-length cDNA of CBL1-like gene in *Sedirea japonica* (*SjCBL1*). Bimolecular fluorescence complementation (BiFC) and pull-down assays demonstrated that like Arabidopsis CBL1 (AtCBL1), SjCBL1 can interacts physically with Arabidopsis CBL-interacting protein kinase 1 (AtCIPK1) at the plasma membrane of plant cells in a Ca^2+^-dependent manner. In addition, overexpression of SjCBL1 in the Arabidopsis *cbl1* mutant resulted in not only rescuing the hypersensitive phenotype toward salt and osmotic stresses, but also substantially enhancing the tolerance to them. Taken together, these results strongly suggest that SjCBL1 is a functional ortholog of AtCBL1 in *Sedirea japonica*, which can play a critical role in response to salt and osmotic stresses. Therefore, it is clear that our findings should significantly contribute to broadening and deepening our understanding of the CBL1-mediated Ca^2+^ signaling networks in the plant kingdom.

## Introduction

Plants must sense and respond to environmental stresses by properly altering their cellular and physiological status to survive and reproduce in such unfriendly conditions. It is well known that this adaptation process involves two critical cellular components, calcium ion (Ca^2+^) and Ca^2+^-binding proteins. First, each environmental stress is transduced into a distinct Ca^2+^ signature in plant cells, which is constituted of not only the magnitude but also the temporal and spatial parameters of calcium spiking (Evans et al., 2001; Rudd and Franklin-Tong, 2001; Sanders et al., 2002; White and Broadley, 2003; Bose et al., 2011). Such complexity enables the simple cation Ca^2+^ to serve as a versatile second messenger in mediating a wide range of extracellular stimuli (McAinsh and Pittman, 2009). Then, a particular Ca^2+^-binding protein(s) acting as a Ca^2+^ sensor molecule perceives the stimulus-specific Ca^2+^ signature and initiates signaling cascades to give rise to an appropriate response. This is possible because plants possess many Ca^2+^-binding proteins with different characteristics in terms of Ca^2+^-binding affinity, subcellular localization, interaction partners, and expression patterns (Weinl and Kudla, 2009; Batistic and Kudla, 2012).

Genomic analysis of the Arabidopsis model plant revealed a number of distinct Ca^2+^ sensor proteins harboring the canonical EF-hand Ca^2+^-binding motifs, which can be classified into four families; 7 calmodulins (CaMs), 50 CaM-like proteins (CMLs), 34 Ca^2+^-dependent protein kinases (CDPKs), and 10 calcineurin B-like (CBL) proteins (Cheng et al., 2002; Kolukisaoglu et al., 2004; McCormack et al., 2005). Based on the modes of decoding Ca^2+^ signals, these EF-hand Ca^2+^ sensors can be divided into two groups, sensor responders and sensor relays (Sanders et al., 2002). The CDPK family belongs to sensor responders, because they have a serine-threonine protein kinase domain (responder) modulated by an intramolecular Ca^2+^-binding domain (sensor) at the C-terminus. On the other hand, the rest of the Ca^2+^-binding protein families belong to sensor relays which do not possess any enzymatic activities themselves and function by interacting with and modulating their target proteins in a Ca^2+^-dependent manner (Luan et al., 2002; Yang and Poovaiah, 2003; Burstenbinder et al., 2017). The CaM and CML family members represent the best examples of sensor relays in that upon Ca^2+^ binding, they undergo conformational changes and regulates various target proteins such as kinase, channel proteins, metabolic enzymes, transcription factors, and so on (Kim et al., 2002; Yang and Poovaiah, 2003; Park et al., 2005; Dobney et al., 2009; Oh et al., 2012; Burstenbinder et al., 2017).

The CBL family is the most lately identified sensor relays, which exhibits some similarity to the regulatory B subunit of the protein phosphatase calcineurin in animals (Liu and Zhu, 1998; Kudla et al., 1999). This calcium sensor proteins physically interact with and activate a group of serine-threonine protein kinases called CIPKs (for CBL-Interacting Protein Kinases) in the presence of Ca^2+^ (Shi et al., 1999; Halfter et al., 2000; Li et al., 2006; Xu et al., 2006; Chaves-Sanjuan et al., 2014). The CIPK family consists of 26 members in Arabidopsis, and each of them is targeted by different subset of the CBL members (Kolukisaoglu et al., 2004; Kimura et al., 2013). It is well known that distinct CBL-CIPK complexes play a role in mediating specific Ca^2+^ signals elicited by a wide variety of environmental stresses such as cold, drought, high salinity, high pH, low K^+^ concentrations, low nitrate condition, and pathogen attacks (Kim, 2013; Yu et al., 2014).

To date, CBL1 is the most extensively studied member of the Arabidopsis CBL family. Analyses of the CBL1 knock-out mutant plants and overexpression lines demonstrated that CBL1 plays a crucial role in plant responses to several abiotic stimuli such as cold, drought, salt, and osmotic stress (Albrecht et al., 2003; Cheong et al., 2003). It is notable that CBL1 targets different members of the CIPK family in order to mediate stimulus-specific responses. For example, CBL1 interacts with CIPK1 to mediate osmotic stress response, whereas it physically associates with CIPK24 to confer salt tolerance (Shi et al., 1999; Kolukisaoglu et al., 2004; D′Angelo et al., 2006). In addition, CBL1 contributes to plant responses to cold and low K^+^ stresses by forming a distinct complex with CIPK7 and CIPK23, respectively (Li et al., 2006; Xu et al., 2006; Huang et al., 2011).

Although significant progress has been made in Arabidopsis CBLs (AtCBLs), their functions in other plant species are still quite limited and inadequate. It is particularly true for the plants that do not have available genome sequences. In the present study, we have developed an efficient way to isolate CBL1-like genes from plants without genomic sequence information. Using this method, we successfully cloned a full-length cDNA of CBL1-like gene from *Sedirea japonica* (*SjCBL1*), which is a small epiphytic orchid plant found mainly in Korea and Japan. This work will definitely expedite the identification of CBL1-like genes in other plants that have not yet been sequenced, and thereby dramatically facilitate our understanding of CBL1-mediated Ca^2+^ signaling networks in the plant kingdom.

## Materials and Methods

### Plant Materials and RNA Preparation

*Sedirea japonica* plants were provided by Dr. Chul Gu Been at Gyeongsangnam-do Agricultural Research & Extension Services (Changwon, Korea). *Arabidopsis thaliana* (ecotype Wassilewsklja [Ws]) plants were grown at 23°C in a growth chamber under long-day conditions (16-h-light/8-h-dark cycle) to the flowering stage for plant transformation. Total RNAs were extracted from plant tissues using the TRIzol reagent (Invitrogen) according to the manufacturer’s instruction. Quality and quantity of the RNAs were monitored by spectrophotometry (OD: 260/280) and by electrophoresis in a 1.2% agarose gel.

### Rapid Amplification of cDNA Ends

5′- and 3′-RACE experiments were performed basically as described by Frohman et al. (1988). First, total RNA prepared from the *Sedirea japonica* leaf and flower tissues was used for first-strand cDNA synthesis. A reaction mixture (14 μL) containing 2 μg of the total RNA and 50 pmol of the mQ024 primer in a 0.5-ml microcentrifuge tube was heated at 70°C for 10 min and quickly chilled on ice. To the chilled mixture, 2 μL of 10X synthesis buffer (200 mM Tris-HCl (pH 8.4), 500 mM KCl, and 25 mM MgCl_2_), 2 μL of 0.1 M DTT, 1 μL of 10 mM dNTP mix and 1 μL of SuperScript II reverse transcriptase (Invitrogen) were added and incubated at 25°C for 10 min. The sample tube was then placed in a 42°C water bath for 50 min and transferred to a 70°C water bath for 15 min. Next, 1 μL of RNase H (2U/μL) was added to the cDNA synthesis reaction and incubated for 20 min at 37°C. Finally, in order to amplify the 3′ end of the SjCBL1 transcript, 2 μL of the cDNA solution were transferred into a fresh 0.5-ml tube containing 1 μL of 10 μM MDP1 and 1 μL of 10 μM mQ024 primers along with 39.5 μL of distilled water, 5 μL of 10X PCR buffer [200 mM Tris-HCl (pH 8.4), 500 mM KCl, and 15 mM MgCl_2_], 1 μL of 10 mM dNTP mix, and 1 μL of Taq DNA polymerase (5U/μL). This mixture was then cycled 30 times in a thermal cycler as follows: 94°C for 30 s; 58°C for 1 min; 72°C for 2 min; with a final 72°C extension of 5 min. The resulting PCR product was subjected to re-amplification with the nested primer MDP2 and mQ025. The re-amplified PCR product was gel electrophoresed, purified, and sequenced. Next, based on the DNA sequence of the 3′-RACE product, nested gene-specific primers, GSP1 and GSP2, were designed in the direction of the unknown 5′-end sequence for 5′-RACE approach. Using these reverse primers and a commercial 5′-RACE kit (Invitrogen), the 5′ end of the SjCBL1 transcript was PCR amplified.

### Construction of Phylogenetic Trees

Evolutionary analyses were conducted in MEGA7 (Kumar et al., 2016). The evolutionary history was inferred using the Minimum Evolution method (Rzhetsky and Nei, 1992) and the confidence probability (multiplied by 100) was estimated using the bootstrap test (500 replicates) (Dopazo, 1994). Using the Poisson correction method (Zuckerkandl and Pauling, 1965), the evolutionary distances were computed and the Minimum Evolution tree was searched with the Close-Neighbor-Interchange (CNI) algorithm (Nei and Kumar, 2000) at a search level of 1. The Neighbor-joining algorithm (Saitou and Nei, 1987) was used to generate the initial tree.

### Yeast Two-Hybrid Assays

A Gal4p-based system (Chien et al., 1991) was used in yeast two-hybrid assays. Genes of interest were cloned into either the DNA-binding domain (pGBT9.BS) or the activation domain (pGAD.GH) vectors. The two plasmids were then introduced into yeast strain Y190 by the lithium acetate method (Schiestl and Gietz, 1989). Yeast cells carrying both plasmids were selected on synthetic medium lacking Leu and Trp (SC-LW) by incubating for 3 days at 30°C. Subsequently, the yeast transformants were streaked on SC-His-Leu-Trp (SC-HLW) plate supplemented with 25 mM 3-amino-1, 2, 4-aminotriazole to determine expression of the *HIS3* nutritional reporter gene. The His^+^ colonies were further analyzed for the expression of β-galactosidase by the filter-lift assay as described previously (Ok et al., 2005).

### Subcellular Localization of GFP-Fusion Proteins and Bimolecular Fluorescence Complementation Assays

The fluorescent protein constructs were introduced into the onion (*Allium cepa*) and tobacco (*Nicotiana benthamiana*) epidermal cells via particle bombardment (Oh et al., 2008) and *Agrobacterium* infiltration (Ok et al., 2015), respectively. The plant cells transiently expressing the fluorescence proteins were analyzed with a confocal laser scanning microscope (LSM 510 META; Carl Zeiss) and Zeiss LSM 510 software (Zeiss LSM Image Examiner). Nuclei were stained with 1 μg mL^-1^ 4′, 6-diamidino-2-phenylindole (DAPI; Sigma) in phosphate-buffered saline solution for 5 min at 23°C and captured with a 417- to 477-nm band-pass filter. For GFP imaging, excitation was performed with an argon laser at 488 nm, and emission was detected with a 515- to 565-nm band-pass filter. YFP excitation was performed with an argon laser at 514 nm, and emission was captured with a 530- to 600-nm band-pass filter.

### Expression and Purification of Glutathione S-Transferase Fusion Proteins

Recombinant GST-fused proteins, such as GST-SjCBL1, GST-SjCBL1:HA, and GST-CIPK1, were expressed and purified basically according to the protocols described previously (Shi et al., 1999). Briefly, *Escherichia coli (E. coli)* BL21 cells carrying each of the GST fusion construct were cultured at 37°C overnight and were subcultured until OD_600_ was 0.5 to 0.6. To induce protein expression, Isopropyl-β-D-thiogalactopyranoside was added to a final concentration of 30 μM. Following 3-h induction at 20°C, the bacterial cultures were collected and ruptured by sonication in ice-cold lysis buffer (50 mM Tris-HCl, pH 7.4, 100 mM NaCl, 1 mM PMSF, 5 mM DTT, 5 mM EDTA, and 1 mM EGTA). Glutathione-Sepharose 4B beads were used to retrieve the GST-fusion proteins from the cell lysate. The beads were washed three times with ice-cold washing buffer (50 mM Tris-HCl, pH 7.4, 100 mM NaCl). Protein concentration was measured with the Bradford assay (Bradford, 1976).

### Gel-Shift and Pull-Down Assays

SjCBL1 and SjCBL1 tagged with a hemagglutinin epitope (SjCBL:HA) proteins were respectively prepared from the recombinant GST-SjCBL1 and GST-SjCBL1:HA proteins by removing GST through thrombin digestion. The purified GST and SjCBL1 proteins were incubated in the washing buffer containing either 5 mM EGTA or 5 mM CaCl_2_ for 1 h on ice and resolved by SDS-PAGE. The protein mobility patterns were visualized with Coomassie blue staining. Pull-down assays were performed as described previously with minor modifications (Shi et al., 1999). Briefly, GST and GST-CIPK1 proteins attached to the glutathione-Sepharose 4B beads were respectively incubated at 4°C for 1 h with the SjCBL1:HA prey protein in the binding buffer (50 mM Tris-HCl, pH 7.4, 100 mM NaCl, 0.05% Tween 20, and 1 mM PMSF) supplemented with either 1 mM CaCl_2_ or 2 mM EGTA. The beads were then centrifuged and washed six times with 500 μl of the corresponding binding buffer. Pull-down samples were resolved by SDS-PAGE and transferred onto polyvinylidene fluoride membranes (Immobilon-P; Millipore) to detect the SjCBL1:HA protein via immunoblotting. Because the recombinant SjCBL1 protein is tagged with the HA epitope, rabbit anti-HA and goat anti-rabbit IgG (H+L) conjugated with horseradish peroxidase (Invitrogen) were used as the primary and secondary antibodies, respectively.

### Generation of Transgenic Plants

For complementation of the *cbl1* mutant (Cheong et al., 2003), the pCAM35S-SjCBL1 construct was transformed into Agrobacterium strain GV3101 and introduced into the Arabidopsis mutant plants by the floral-dip method (Clough and Bent, 1998). Seeds were harvested from these plants and screened on 1/2 MS medium containing 0.8% (w/v) agarose, 112 mg/L Gamborg’s B5 vitamin mixture, 50 μg/mL kanamycin, and 15 μg/mL hygromycin. The selected seedlings were transplanted to soil and grown in a greenhouse to produce seeds. Homozygous complemented lines (denoted *cbl1/SjCBL1*) were isolated and used for further analysis.

### Quantitative Reverse Transcriptase-Mediated PCR (RT-PCR) Analysis

To analyze the expression levels of *SjCBL1* in the transgenic plants by RT-PCR, first strand cDNA was reverse-transcribed from 2 μg of total RNA using 0.5 μg oligo (dT) and 200 units of SuperScript II reverse transcriptase (Invitrogen) according to the manufacturer’s instructions. One microliter of cDNA synthesized in the reverse transcription reaction was used as template for a 25-μL PCR reaction with 10 pmol of gene-specific primers (SjCBL1 forward, 5′-GTCATTGATTTTGGTGACTTCGTTC-3′, SjCBL1 reverse, 5′-ATGATGGGTTGCGAGAAACAAAATT-3′). The eukaryotic translation initiation factor 4A1 (EIF4A1, AT3G13920) gene was also amplified by a pair of primers (forward, 5′-GCTCTCCCGTGGTTTCAAGGACCAG-3′; reverse, 5′-GTCTGTGAGCCAATCAACCTTACGC–3′) and used as a quantitative control for *Arabidopsis thaliana*. In the case of *Sedirea japonica*, SjActin (GenBank accession number: JN981139.1) gene was employed as an internal normalization control by using the following primer set (SjActin-F, 5′-GAAGTTGCAGGCATTCATGAGACC-3; SjActin-R, 5′-CTATATTTCCTCTCAGGTGGGGC-3). To investigate the *CBL1* expression in Ws and *cbl1* plants, the following gene-specific primer set was used; CBL1 forward, 5′-GATCGCGCTTCTCTGCGAATCTGAA -3′ and CBL1 reverse, 5′-CAGTTTGTTTCTTCATGTGGCAATC-3′. PCR amplification was performed with initial denaturation at 94°C for 2 min followed by 26 cycles of incubation at 94°C for 30 s, 56°C for 30 s, and 72°C for 30 s, with a final extension at 72°C for 5 min. Five microliters out of 25-μL reactions were resolved on a 1.2% (w/v) agarose gel, stained with ethidium bromide, and visualized by UV light.

### Germination Assay and Salt Tolerance Analysis

Approximately 100 seeds each from the wild-type (Ws), the *cbl1* mutant, and the *cbl1*/*SjCBL1* complemented line were planted in triplicate on MS medium supplemented with different concentrations of NaCl or mannitol and incubated at 4°C for 3 days before being placed at 23°C under long-day conditions (16-h-light/8-h-dark cycle). Germination (radicle emergence) was scored daily for 7 days. For salt tolerance analyses, 4-day old seedlings were transferred to MS agar medium supplemented with 125 mM NaCl and grown for 10 days in a vertical position. In addition, 3-week old plants in soil were exposed to 150 mM NaCl solution every 3 days for four repetitions.

### Chlorophyll Measurement

The chlorophyll content in leaves was determined as described by Strain et al. (1971). Briefly, the Arabidopsis leaves were harvested and homogenized in liquid nitrogen. To extract chlorophyll, 3 volumes of 80% (v/v) acetone containing 1 μM KOH was added to the ground tissue and centrifuged at 16,000 *g* for 2 min. Then, supernatants were subject to spectrophotometric analysis.

### Construction of Plasmids

The following plasmids were created as described previously (Shi et al., 1999; Walter et al., 2004); pGBT⋅CIPK1, pGBT⋅K292, pGBT⋅C169, pGAD⋅CIPK1, pGEX⋅CIPK1, bZIP63-YFP^N^, and bZIP63-YFP^C^. The five plasmids from pGAD⋅CIPK2 to pGAD⋅CIPK6 were described in Kim et al. (2000), and the nineteen plasmids from pGAD⋅CIPK7 to pGAD⋅CIPK25 were produced as described previously (Kolukisaoglu et al., 2004). The pGAD⋅CIPK26 construct was made by cloning the coding region of *AtCIPK26* cDNA, amplified with CIPK26-1 and CIPK26-2 primers, into the into *EcoR*I/*Sal*I sites of pGAD.GH. To create the pGBT⋅C152, pGBT⋅C133, and pGBT⋅C54 plasmids, the corresponding CIPK1 regions were PCR amplified with primer sets, CIPK1-8/CIPK1-2, CIPK1-9/CIPK1-2, and CIPK1-16/CIPK1-2, respectively and cloned into the *Eco*RI/*Sal*I sites of the pGBT9⋅BS vector. To construct pGBT⋅SjCBL1 and pGAD⋅SjCBL1 plasmids, the coding region of the *SjCBL1* cDNA was PCR amplified with a primer set (SjCBL1-1 and SjCBL1-2) using 2 μL of the *Sedirea japonica* cDNAs prepared above as template. The PCR product was digested with *Eco*RI and *Sal*I restriction enzymes and then ligated into the pGBT9.BS and pGAD.GH vectors, respectively.

For creation of the SjCBL1-GFP fusion construct (pCAM⋅SjCBL1-GFP), a primer set of SjCBL1-3 and SjCBL1-4 was used to amplify the SjCBL1 coding region without a stop codon. Following digestion with *Bgl*II/*Spe*I, the resulting PCR product was cloned into the pCAMBIA1304 binary vector that contains a GFP reporter gene (Cambia, Australia). For BiFC assays in onion epidermal cells, the SjCBL1 coding region lacking a stop codon was amplified with SjCBL1-5 and SjCBL1-6 primers and ligated into the *Spe*I/*Sal*I, sites of pUC-SPYCE vector, producing pUC-SPYCE⋅SjCBL1 (SjCBL1-YFP^C^) plasmid. In a similar way, pUC-SPYNE⋅CIPK1 (CIPK1-YFP^C^) was generated by cloning the CIPK1 PCR product, amplified with CIPK1-14 and CIPK1-23 primers, into the *Xba*I/*Bam*HI sites of pUC-SPYNE vector (Walter et al., 2004).

To perform multicolor BiFC assays in tobacco epidermal cells, the plant binary vectors pVYNE and pMAS⋅SCYCE(R) were used (Waadt et al., 2008). To make pVYNE⋅SjCBL1 (SjCBL1-VENUS^N173^), a primer set of SjCBL1-7 and SjCBL1-6 was used to amplify the SjCBL1 coding region. After digestion with *Bgl*II/*Sal*I, the resulting PCR fragment was ligated into the *Bam*HI/*Sal*I sites of pVYNE. To construct pMAS-SCYCE(R)⋅CIPK1 (SCFP3A^C155^-CIPK1), the pUC-SPYNE⋅CIPK1 plasmid was digested with *Xba*I and *Bam*HI and the CIPK1 insert was ligated into the *Spe*I/*Bam*HI sites of pMAS-SCYCE(R) vector.

The GST-SjCBL1 fusion construct (pGEX⋅SjCBL1) was generated by cloning the PCR product amplified with SjCBL1-7 and SjCBL1-6 primers into the *Bgl*II/*Sal*I sites of pGEX-4T-3 (GE Healthcare Life Sciences). In addition, the SjCBL1:HA region in the pUC-SPYCE⋅SjCBL1 plasmid was amplified with SjCBL1-7 and HA-R primers to construct pGEX⋅SjCBL1:HA plasmid. The PCR product was digested with *Bgl*II/*Not*I and cloned into the *Bam*HI/*Not*I sites of the pGEX-4T-3 vector. To express the SjCBL1 protein in the *cbl1* mutant, the SjCBL1 cDNA was placed under the control of 35S promoter in the pCAMBIA1300 binary vector (Cambia, Australia). This chimeric construct was designated pCAM35S⋅SjCBL1 and was generated as follows. The coding region of the SjCBL1 cDNA was amplified with a primer set of SjCBL1-5 and SjCBL1-8. The resulting PCR product was digested with *Spe*I/*Bgl*II and cloned into the *Xba*I/*Bam*HI sites of the pCAM35S binary vector (Cho et al., 2016). All PCRs were carried out using *Pfu* DNA polymerase (Stratagene) to enhance fidelity. All of the constructs above were verified by DNA sequencing.

### Oligonucleotide Primers

Primers used in this study for cloning were listed below. Restriction enzyme sites were underlined. Three additional bases were included at the 5′ end of the primers for efficient restriction enzyme digestion:

mQ024: 5′-GGTCGACTCGAGTCGACATCGATTTTTTTTTTTTTTTTT-3′:
mQ025: 5′-GTGCGACTCGAGTCGACATCGA-3′:
MDP1: 5′-GCW(A+T)Y(C+T)TR(A+G)TTY(C+T)GAGCTR(A+G)TTCAAG-3′;
MDP2: 5′-R(A+G)TTY(C+T)GAGCTR(A+G)TT CAA GAG CAT-3′;
GSP1: 5′-AGTTGTGATGTCCTTGAGATATTG-3′;
GSP2: 5′-TGATGTCCTTGAGATATTGAAGTG-3′;
SjCBL1-1: 5′-TTAGAATTCAATGGGCTGCTTTCACTCTA-3′;
SjCBL1-2: 5′-AAAGTCGACTCAAGTAGCTATGTCATCAA-3′;
SjCBL1-3: 5′-AAAAGATCTAATGGGCTGCTTTCACTCTA-3′;
SjCBL1-4: 5′-AAAACTAGTAGTAGCTATGTCATCAACCT-3′;
SjCBL1-5: 5′-AAAACTAGTATGGGCTGCTTTCACTCTAA-3′;
SjCBL1-6: 5′-AAAGTCGACAGTAGCTATGTCATCAACCT-3′;
SjCBL1-7: 5′-AAAAGATCTATGGGCTGCTTTCACTCTAA-3′;
SjCBL1-8: 5′-AAAAGATCTAGTAGCTATGTCATCAACCT-3′;
HA-R: 5′-AAAGCGGCCGCAGCGTAATCTGGAACATCGT-3′;
CIPK1-2: 5′-AAAGTCGACCTAAGTTACTATCTCTTGCT-3′;
CIPK1-8: 5′-TAAGAATTCAAGTGATTCACCGACCATCA-3′;
CIPK1-9: 5′-TAAGAATTCATTTGAACAAGAGAATGTAT-3′;
CIPK1-16: 5′-TAAGAATTCACAAGTTGGTTTATCAGTAA-3′;
CIPK1-14: 5′-ATATCTAGAATGGTGAGAAGGCAAGAGGA-3′;
CIPK1-23: 5′-TTTGGATCCAGTTACTATCTCTTGCTCCG-3′;
CIPK26-1: 5′-ATTGAATTCGATGAATCGGCCAAAGGTTC-3′
CIPK26-2: 5′-CTTGTCGACTTAGACCAGAGCTCTCGCCA-3′

## Results

### Molecular Cloning and Sequence Analysis of SjCBL1

**FIGURE 1:**
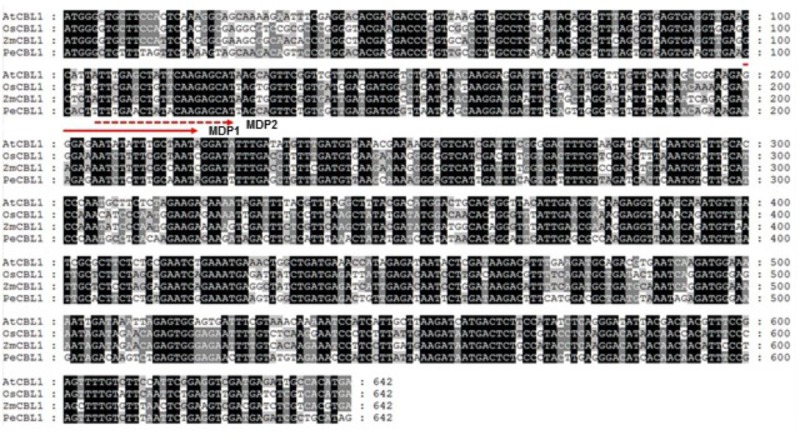
Sequence alignment of CBL1 cDNAs from various plants. Nucleotide sequences of OsCBL1 (GenBank accession No. DQ201195), ZmCBL1 (EU907931), PeCBL1 (DQ337190) and AtCBL1 (AT4G17615) were aligned using Lasergene MegAlign Program (DNASTAR) and visualized with GeneDoc version 2.7. Nucleotides conserved in all four plant species are shaded black, and nucleotides conserved in 2–3 plants are shaded gray. The dashed and solid lines with an arrow indicate regions used to design two forward degenerative primers (MDP1 and MDP2) for 3′RACE.

Following the initial discovery of CBLs from Arabidopsis (Liu and Zhu, 1998; Kudla et al., 1999), several CBL-like proteins have also been isolated and characterized from various plant species with available genome sequences, including rice, pea, maize, and poplar (Kolukisaoglu et al., 2004; Mahajan et al., 2006; Wang et al., 2007; Zhang et al., 2013; Tang et al., 2014). Therefore, it is believed that the CBL-type calcium sensor proteins are ubiquitously present in the plant kingdom (Batistic and Kudla, 2009). However, little is known about their functions in *Sedirea japonica*, mainly because the genome sequence of the orchid plant has not yet been determined. Here, we have developed a novel method for isolating a CBL1-like gene in *Sedirea japonica* (SjCBL1) without knowing the genomic sequence: Comparing the nucleotide sequences of CBL1 cDNAs identified in several plants such as Arabidopsis, rice (*Oryza sativa*), maize (*Zea mays*), and poplar (*Populus euphratica*), we were able to find a highly conserved region and design two moderately degenerate primers, MDP1 and MDP2, which can anneal to the evolutionarily conserved motif (**Figure 1**). Using these primers and total RNAs prepared from the leaves and flowers of *Sedirea japonica*, we performed 3′ RACE and successfully obtained a cDNA fragment containing the 3′ end of SjCBL1.

**FIGURE 2:**
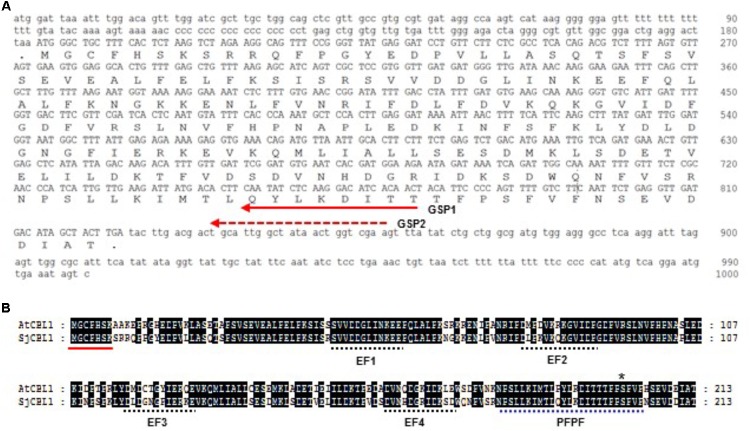
Sequence analysis of the SjCBL1 cDNA clone. **(A)** Nucleotide sequence of the isolated SjCBL1 cDNA. Open reading frame is shown in uppercase, whereas the 5′ and 3′ untranslated regions are indicated in lowercase. The dashed and solid lines with an arrow indicate two nested reverse primers (GSP1 and GSP2) used for 5′RACE. **(B)** Amino acid sequence alignment of Arabidopsis CBL1 (AtCBL1) and *Sedirea Japonica* CBL1 (SjCBL1). Alignment was generated by using Lasergene MegAlign Program (DNASTAR) and visualized with GeneDoc version 2.7. Identical amino acid residues are shaded black. A solid line indicates the canonical myristoylation motif (MGXXXSK) and dashed lines represent the EF-hand or PFPF motifs. The asterisk mark represent the serine residue phosphorylated by CIPK23.

Based on the nucleotide sequence of the 3′ RACE product, we made two nested gene-specific primers, GSP1 and GSP2, and used them to isolate the 5′ end of SjCBL1 via 5′ RACE. As a result, we were able to come up with the full-length cDNA sequence of the SjCBL1 gene (GenBank accession no. MG867729), which contains a 642-bp open reading frame (ORF) encoding a polypeptide of 214 amino acid residues (**Figure 2A**). Amino acid sequence analysis revealed that SjCBL1 shares 78% identity (92% similarity) with the *Arabidopsis* CBL1 (AtCBL1) and contains the *N*-myristoylation motif (MGXXXS/T) as shown in **Figure 2B**, which mediates membrane association and protein-protein interaction in Arabidopsis plant cells (Batistic et al., 2008). Like AtCBL1, SjCBL1 also possesses four EF-hand Ca^2+^ -binding motifs (Kawasaki et al., 1998; Akaboshi et al., 2008) and the C-terminus PFPF motif that is found only in CBL family members (Du et al., 2011).

**FIGURE 3:**
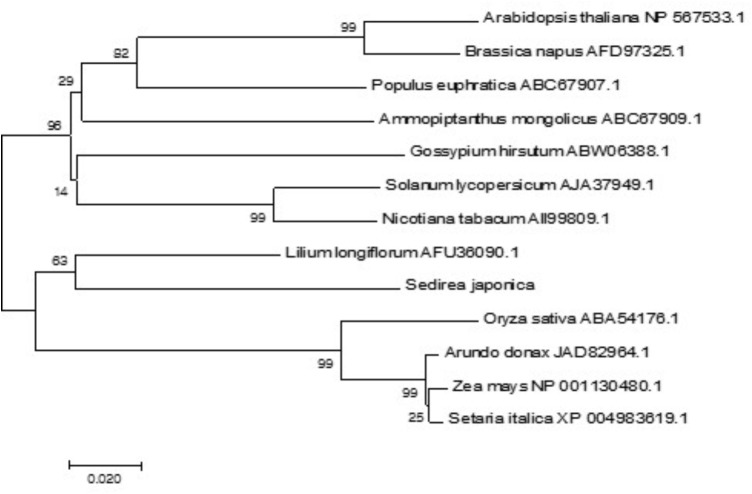
Phylogenetic relationships of CBL1 genes in plants. Alignment of 13 full-length protein sequences and phylogenetic analyses were performed as described in “Materials and methods.” The accession numbers of the CBLs are presented next to the species names. The optimal tree with the sum of branch length = 0.83257379 is shown and bootstrap values are indicated at the respective branches. The tree is drawn to scale, with branch lengths in the same units as those of the evolutionary distances used to infer the phylogenetic tree.

Using the MEGA7 software program, we constructed a phylogenetic tree for 13 CBL1 homologues from different plant species on the basis of the deduced amino acid sequences. As shown in **Figure 3**, the phylogenetic analysis indicated that SjCBL1 is most related to the Easter lily (*Lilium longiflorum*) CBL1. Moreover, it also clearly showed the evolutionary relationships among the plants including monocots and dicots, suggesting that CBL1 can be used as a reference gene for evaluating evolutionary distances between plant species.

### SjCBL1 Interacts With Multiple Members of the Arabidopsis CIPK Family

Sequence analysis showed that SjCBL1 is very similar to AtCBL1 at least on the amino acid level. Therefore, we decided to first investigate whether SjCBL1 can interact with AtCIPK1, the most thoroughly studied AtCBL1 interactor. To this end, full-length cDNAs of SjCBL1 and AtCIPK1 were cloned in the DNA-binding domain (pGBT⋅BS; BD) and GAL4 activation domain (pGAD⋅GH; AD) vectors, respectively, producing pGBT⋅SjCBL1 (BD⋅SjCBL1) and pGAD⋅AtCIPK1 (AD⋅CIPK1). Following transformation of the yeast strain Y190 cells with these BD and AD constructs, interaction between the two proteins was monitored by analyzing expression of the reporter genes, imidazole glycerol-phosphate dehydratase (*HIS3*) and β-galactosidase (*LacZ*).

**FIGURE 4:**
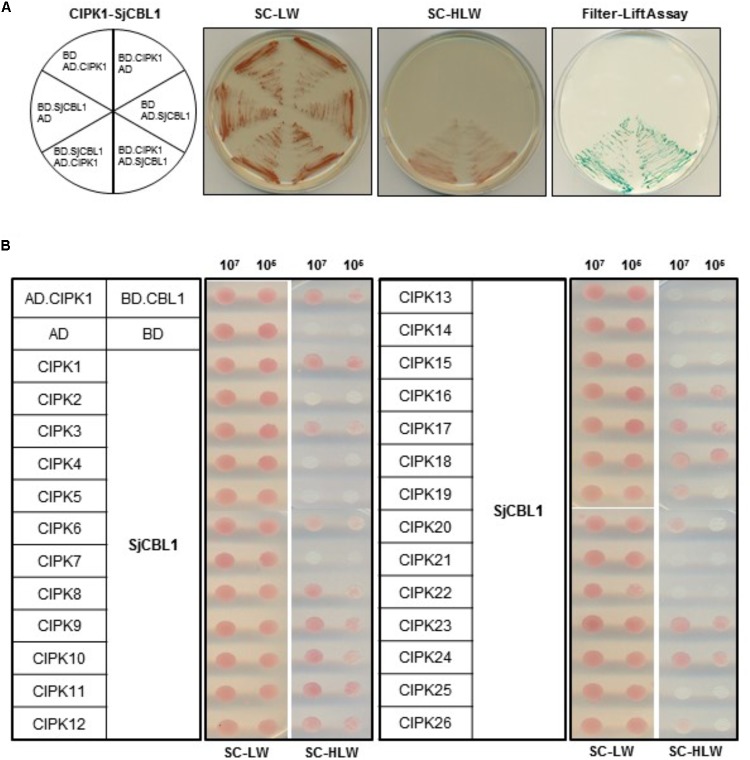
A yeast two-hybrid assay showing that SjCBL1 interacts with multiple members of AtCIPK family. **(A)** SjCBL1 interacts with AtCIPK1 in a vector-dependent manner. The first panel at left shows the arrangement of the Y109 yeast cells harboring the indicated BD and AD plasmids. The second and third panels display yeast growth on synthetic complete medium lacking Leu and Trp (SC-LW) and synthetic complete medium lacking His, Leu, and Trp (SC-HLW), respectively. The last panel shows *β*-galactosidase activity using filter-lift assay. **(B)** Comparative interaction analysis of SjCBL1 with 26 members of the Arabidopsis CIPK family. The yeast cells were cotransformed with the combinations of the BD and AD constructs as indicated at the left. Cotransformed yeast cells were then cultured, serially diluted, and spotted onto the indicated media.

As shown in **Figure 4A** (the left half circle), the yeast transformants possessing both BD⋅SjCBL3 and AD⋅CIPK1 grew well on the selection medium (SC-HLW) and developed a blue color in the filter lift assay, indicating expression of the HIS3 and LacZ genes. However, the control yeast cells harboring either BD⋅SjCBL1/AD or BD/AD⋅CIPK1 plasmid combinations failed to express the reporter genes. These results indicated that like AtCBL1, SjCBL1 can also interacts with AtCIPK1. In addition, we also constructed AD⋅SjCBL1 and BD⋅CIPK1 plasmids to perform vector-swapping analysis. As shown in **Figure 4A** (the right half circle), the two reporter genes were expressed in the yeast cells possessing AD⋅SjCBL3 and BD⋅CIPK plasmids, demonstrating that the SjCBL1 and AtCIPK1 interaction can take place in a vector-independent manner.

According to the previous yeast two-hybrid assays, AtCBL1 also displayed interaction affinity to other nine AtCIPK members than AtCIPK1 (Kolukisaoglu et al., 2004). Therefore, we further tested if SjCBL1, like AtCBL1, can exhibit similar interaction patterns to the heterologous 26 members of the Arabidopsis CIPK family. All AtCIPK family members were cloned into the GAL4 activation domain vector, producing twenty six AD⋅CIPK plasmids from AD⋅CIPK1 to AD⋅CIPK26. As shown in **Figure 4B**, SjCBL1 and AtCBL1 interacted with AtCIPK1 at the similar strength and shared nine of the 10 interactors such as AtCIPK1, 7, 8, 11, 12, 17, 18, 23, and 24. Only AtCIPK5 failed to interact with SjCBL1 unlike AtCBL1. Interestingly, SjCBL1 also interacted with additional seven AtCIPK members such as AtCIPK3, 6, 9, 10, 16, 19, and 20.

**FIGURE 5:**
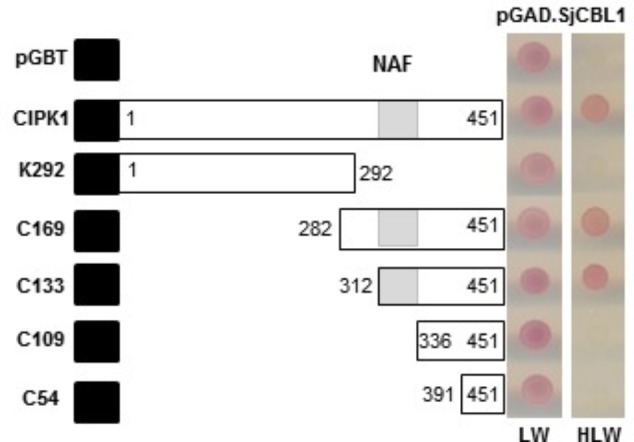
Determination of the CIPK1 region responsible for the interaction with SjCBL1. A series of SjCBL1 deletion mutants were created in the pGBT vector and cotransformed into the Y190 yeast cells with pGAD⋅SjCBL1. Numbers in the white boxes indicate the beginning and the ending positions of each protein fragment. Black and shaded boxes denote the DNA-binding domain of the GAL4 transcription factor and NAF domain, respectively. Two panels on the right display yeast growth on synthetic complete medium lacking Leu and Trp (SC-LW) and synthetic complete medium lacking His, Leu, and Trp (SC-HLW), respectively.

To delimit the AtCIPK region necessary for the interaction with SjCBL1, we chose AtCIPK1 for further study and created a series of deletion constructs by cloning AtCIPK1 fragments into the BD vector. As shown in **Figure 5**, the N-terminal deletions down to 312th amino acid residue of AtCIPK1 (C169 and C133) maintained the ability to interact with SjCBL1, whereas further removal of 23 amino acid residues (C109) carrying the NAF (also known as FISL) motif (Albrecht et al., 2001; Guo et al., 2001) completely abolished the interaction. In addition, the C-terminal deleted form (K292) failed to display such affinity. Therefore, these deletion analyses demonstrated that the AtCIPK1 C-terminal motif between 312th and 336th amino acid residues is required for the SjCBL1-AtCIPK1 interaction.

### SjCBL1 Mainly Localizes at the Plasma Membrane

**FIGURE 6:**
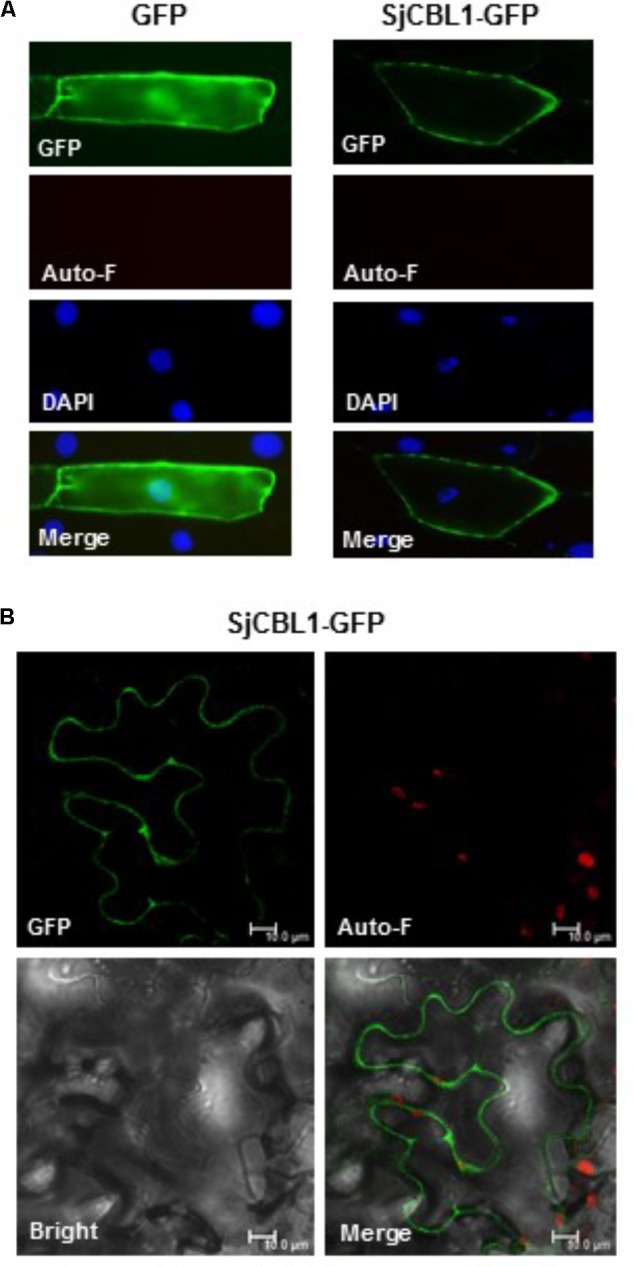
Subcellular localization of SjCBL1-GFP protein in a plant cell. **(A)** Fluorescent images of onion epidermal cells. The indicated plasmids were introduced into the plant cells via the biolistic particle-delivery system. Following a 18-h incubation at 23°C, the onion cells were analyzed with a fluorescence microscope. GFP and Auto-F indicate signals of GFP fluorescence and auto-fluorescence, respectively. DAPI shows nuclei visualized by 4′, 6-diamidino-2-phenylindole (DAPI) staining. The merged images are shown in Merge. **(B)** Tobacco (*Nicotiana benthamiana*) leaf epidermal cells infiltrated with Agrobacterium carrying the SjCBL1-GFP chimeric construct. *Scale bars*, 10 μm.

To determine the subcellular localization of SjCBL1, we first fused green fluorescent protein (GFP) to the C-terminal end of the SjCBL1 protein and created the SjCBL1-GFP fusion construct driven by the cauliflower mosaic virus 35S promoter (pCAM⋅SjCBL1-GFP). We then introduced this chimeric construct into the onion (*Allium cepa*) epidermal cells via the particle bombardments and observed the GFP fluorescence with a confocal laser scanning microscope. As displayed in **Figure 6A**, this transient expression analysis showed that SjCBL1-GFP was mainly localized at the plasma membrane of the onion cell. The GFP control, however, was found almost uniformly throughout the whole cytoplasm. These results clearly indicate that SjCBL1 harbors information to direct the plasma membrane targeting.

Using the tobacco (*Nicotiana benthamiana*) leaves infiltrated with *Agrobacterium tumefaciens* possessing the SjCBL1-GFP construct, we further verified the SjCBL1-GFP localization. As shown in **Figure 6B**, the tobacco cells expressing the SjCBL1-GFP fusion protein also exhibited strong GFP fluorescent signals along the plasma membrane, demonstrating that SjCBL1-GFP is bound to the plasma membrane. Taken together, our results strongly suggest that SjCBL1 functions at the plasma membrane of the orchid plant cells.

### SjCBL1 Is a Functional Ca^2+^-Binding Protein

**FIGURE 7:**
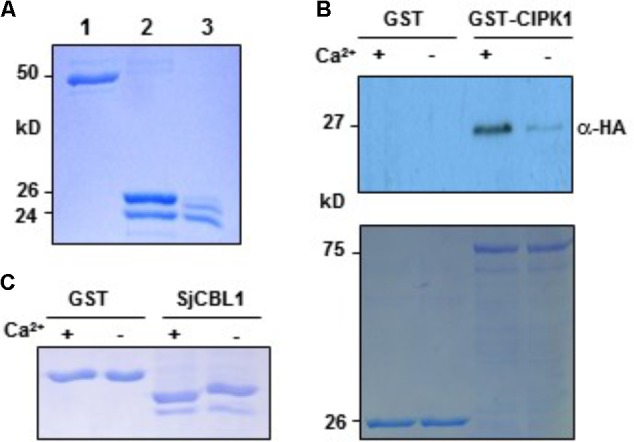
*In vitro* interaction between SjCBL1 and CIPK1 in a Ca^2+^-dependent manner. **(A)** Purification of the recombinant SjCBL1 protein expressed in *Escherichia coli.* Lanes 1–3 contain the GST-SjCBL1 form, the thrombin digested forms of GST and SjCBL1, and purified SjCBL1, respectively. **(B)** Gel shift assay showing Ca^2+^-binding affinity of the recombinant SjCBL1 protein. The indicated proteins were incubated in 2 mM EGTA (–) or 1 mM CaCl_2_ (+) before being analyzed with SDS-PAGE. **(C)** Pull-down assay. The GST-CIPK1 fusion protein was used as a bait to pull down the prey SjCBL10:HA in the presence (+, 1 mM CaCl_2_) or absence (–, 2 mM EGTA) of calcium. For a negative control, GST alone was used as a bait. The top panel shows a western blot probed with rabbit anti-HA antibody and the bottom panel shows a Coomassie Brillant Blue stained SDS-PAGE gel indicating the amount of bait proteins used in each pull-down assay.

Sequence analysis above revealed that SjCBL1 possesses the typical EF-hand Ca^2+^-binding motifs, suggesting that it may act as a Ca^2+^ sensor. We therefore tested if the SjCBL1 protein actually has the ability to bind Ca^2+^ *in vitro*. To this end, we first purified the recombinant SjCBL1 protein produced in *E. coli* using the GST gene fusion system. **Figure 7A** shows an approximately 24-kD SjCBL1 protein band that was originally purified as a GST-fused form (∼50 kD) and subsequently digested with thrombin to remove the GST tag.

We then used this GST-free SjCBL1 protein to perform the gel shift assay in which Ca^2+^-binding proteins migrate at different rates during gel electrophoresis depending on Ca^2+^ availability in the loading buffer (Krinks et al., 1988). As shown in **Figure 7B**, the SjCBL1 protein displayed a faster migration rate in the lane containing Ca^2+^ compared to the lane without Ca^2+^ (chelated with EGTA). In contrast, such mobility shift was not observed in the GST protein used as a negative control. Therefore, it is clear that the SjCBL1 gene encodes a functional Ca^2+^-binding protein.

### SjCBL1 Directly Associates With AtCIPK1 in a Ca^2+^-Regulated Manner

To verify the SjCBL1-AtCIPK1 interaction demonstrated by the yeast two-hybrid assay and to examine whether Ca^2+^ plays a regulatory role in such complex formation, we performed pull-down assays in the absence or presence of Ca^2+^ using the recombinant GST-AtCIPK1 (bait) and HA-tagged SjCBL1 (prey) proteins (see Materials and Methods). As depicted in **Figure 7C**, the Ca^2+^- binding protein SjCBL1 was much more efficiently retrieved by the GST-AtCIPK1 bait protein in the presence of Ca^2+^ than in the absence of Ca^2+^. In contrast, GST alone failed to pull down the prey protein regardless of Ca^2+^. Therefore, it is clear that SjCBL1 physically associates with AtCIPK1, supporting the observation in the yeast two-hybrid assays. Furthermore, these *in vitro* interaction results also suggest that Ca^2+^ can increase the interaction strength between SjCBL1 and AtCIPK1.

### SjCBL1 and AtCIPK1 Physically Interact at the Plasma Membrane of Plant Cells

**FIGURE 8:**
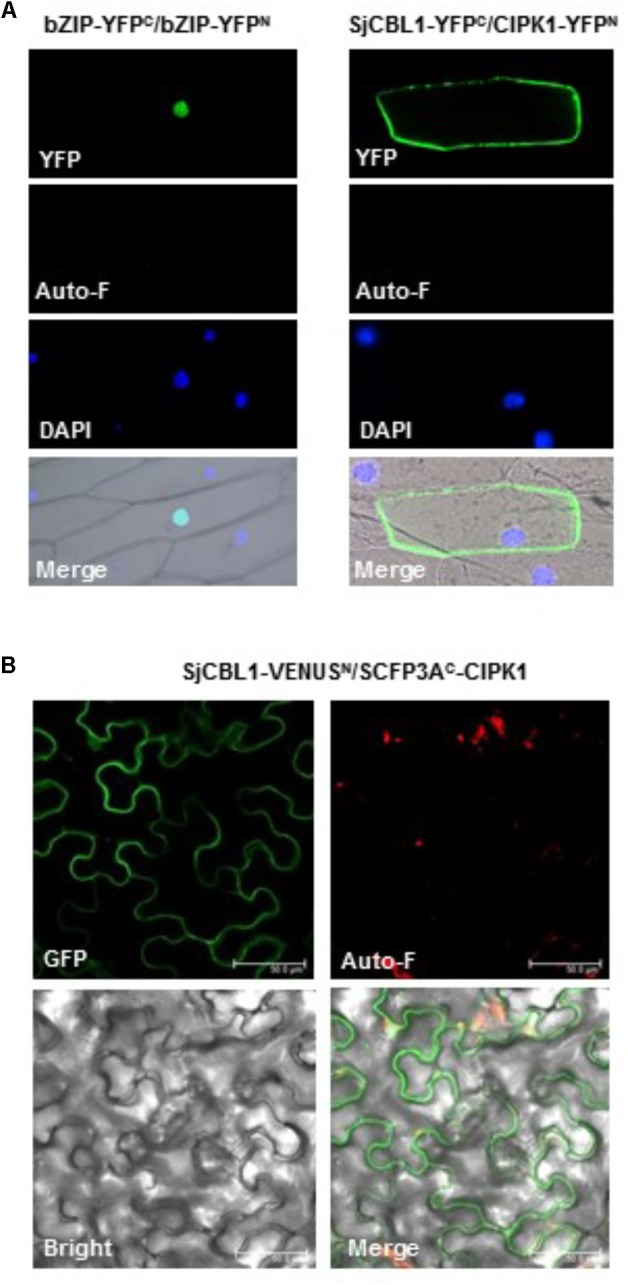
*In vivo* interaction between SjCBL1 and CIPK1. **(A)** Bimolecular fluorescence complementation (BiFC) analysis in onion epidermal cells. The indicated plasmids were introduced into the plant cells via the biolistic particle-delivery system. The bZIP63-YFP^N^ and bZIP63-YFP^C^ plasmids were used as a positive control. Following 18-h incubation at 23°C, the onion cells were analyzed with a fluorescence microscope. YFP and Auto-F indicate images of YFP fluorescence and auto-fluorescence, respectively. DAPI shows nuclei visualized by DAPI staining. The merged images are shown in Merge. **(B)** Multicolor BiFC analysis in tobacco (*Nicotiana benthamiana*) leaf epidermal cells infiltrated with Agrobacterium carrying SjCBL1-VENUS^N173^ and SCFP3A^C155^-CIPK1. GFP, Auto-F, and Bright indicate images of GFP fluorescence, auto-fluorescence, and bright field, respectively. Merge represents the merged image. *Scale bars*, 50 μm.

We further confirmed the physical interaction between SjCBL1 and AtCIPK1 in living plant cells using the BiFC assay (Walter et al., 2004). For this analysis, we generated and introduced the SjCBL1-YFP^C^ and CIPK1-YFP^N^ fusion constructs into onion epidermal cells via particle bombardments. As shown in **Figure 8A**, the onion cells expressing the two fusion proteins displayed strong YFP fluorescence in the plasma membrane, whereas the control cells expressing bZIP63-YFP^N^ and bZIP63-YFP^C^ exhibited the fluorescence only in the nucleus. The Arabidopsis bZIP63 gene (At5g28770) encodes a member of the basic leucine zipper (bZIP) transcription factors that forms a homodimer in the nucleus of plant cells (Siberil et al., 2001). Therefore, these fluorescent images clearly indicate that the SjCBL1 and AtCIPK1 proteins physically associated with each other at the plasma membrane of onion cells.

Furthermore, we also created SjCBL1-VENUS^N173^ and SCFP3A^C155^-CIPK1 plasmids using the plant binary pVYNE and pMAS⋅SCYCE(R) vectors, respectively, and carried out multicolor BiFC assays in tobacco epidermal cells (Waadt et al., 2008). As shown in **Figure 8B**, these chimeric proteins expressed in the tobacco leaf cells via the *Agrobacterium*-infiltration method produced GFP fluorescence signals almost exclusively at the plasma membrane, supporting their physical interaction in the tobacco cells. Taken together, our results strongly suggest that SjCBL1 and AtCIPK1 can form a complex at the plasma membrane of plant cells.

### SjCBL1 Transcript Levels Are Increased by Salt Stress

**FIGURE 9:**
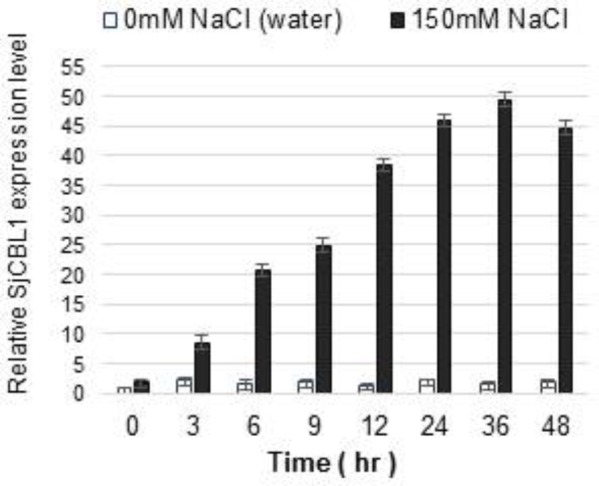
Induction of SjCBL1 expression levels by high salt treatment in *Sedirea japonica*. Quantitative RT-PCR analysis was used to determine the SjCBL1 transcript levels in the orchid plants. Total RNAs were isolated at the indicated time points from the leaves of 7-month-old orchid plants treated with NaCl (150 mM) or water (as a control). An actin gene identified in Sedirea japonica, SjActin (GenBank accession number: JN981139.1), was employed as an internal control for transcript normalization. The SjCBL1 expression level at the 0 time point was set to 1.0 to show relative abundance differences.

We previously demonstrated that expression of the CBL1 gene is induced by high salt stress in Arabidopsis (Cheong et al., 2003). Therefore, we were interested in investigating whether SjCBL1 gene also displays such salt-inducible expression in *Sedirea japonica* plants. Using qRT-PCR analysis, we determined SjCBL1 transcript levels in the leaves of the orchid plants. As shown in **Figure 9**, treatment of 150 mM NaCl solution greatly increased mRNA levels of the *SjCBL1*gene, whereas water control had no effect. This result suggests that, like the Arabidopsis counterpart, SjCBL1 gene is also strongly regulated by high salinity and may play a role in mediating the salt stress response in *Sedirea japonica*.

### Overexpression of SjCBL1 Rescues the Sensitive Phenotype of the Arabidopsis *cbl1* Mutant

**FIGURE 10:**
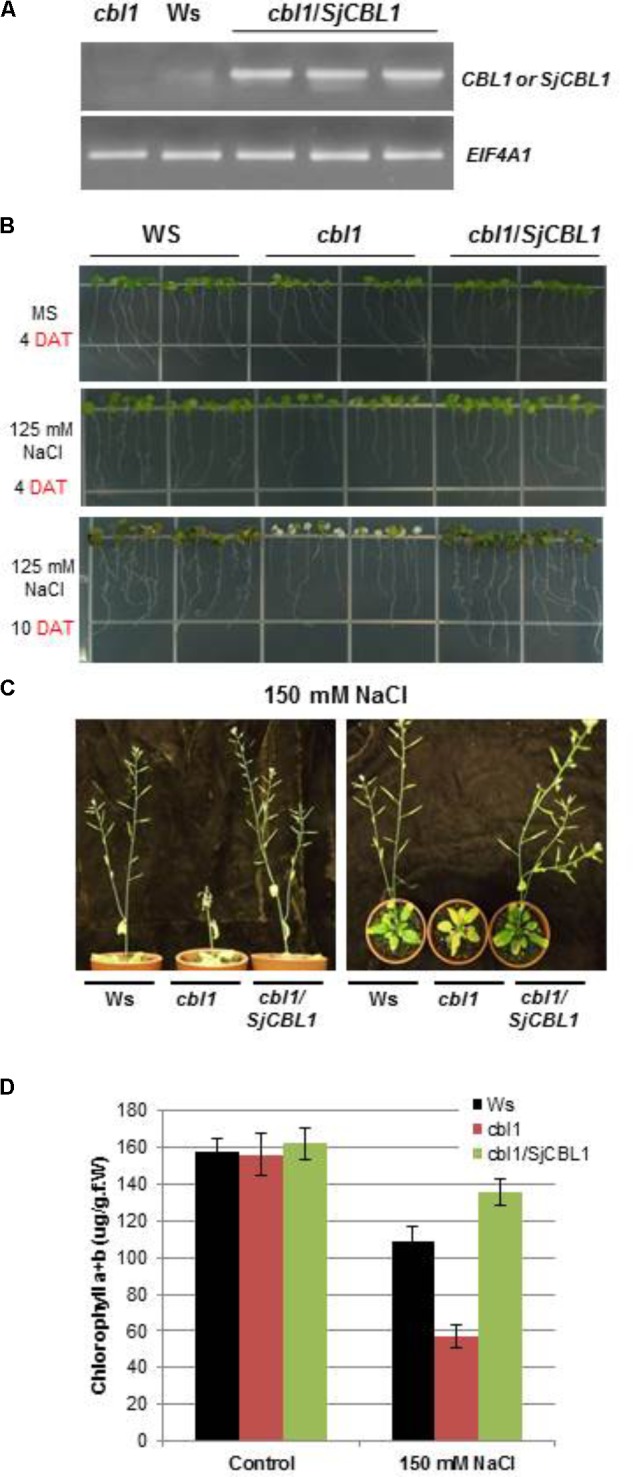
Phenotype rescue in complemented (c*bl1/SjCBL1*) plants under salt stress. **(A)** Quantitative RT-PCR analysis of the CBL1 or SjCBL1 gene expression in wild-type (Wassilewskija [Ws]), *cbl1* mutant, and complemented (c*bl1/SjCBL1*) plants. Total RNAs were isolated from 3-week-old plants and either CBL1-or SjCBL1-specific primers (top gel) were used for PCR amplification. Arabidopsis EIF4A1 gene (AT3G13920) was used as an internal control for transcript normalization (bottom gel). **(B)** Vertical growth assay. Seedlings at 4 days after germination were transferred to MS agar media supplemented with or without 125 mM NaCl, which were then placed vertically in the growth chamber. The pictures were taken on the 4th and 10th days after transfer (DAT). **(C)** Salt stress tolerance of the mature *cbl1/SjCBL1* plants. Three-week old plants were exposed to 150 mM NaCl solution every 3 days for four repetitions. The pictures were taken on the 12th day after treatment from the side (left panel) and top (right panel). **(D)** Chlorophyll content of the plants from the experiment described in **(C)**.

The Arabidopsis *cbl1* mutant plants lacking functional AtCBL1 protein are less tolerant to salt and osmotic stress conditions than the wild-type (Wassilewskija [Ws] plants, Cheong et al., 2003). To examine the biological function of the SjCBL1 gene, we transformed the null mutant with a chimeric construct of SjCBL1 cDNA driven by CaMV 35S promoter (pCAM35S⋅SjCBL1) and obtained several independent transgenic plants. As depicted in **Figure 10A**, these complementation lines (*cbl1/SjCBL1*) exhibited higher levels of SjCBL1 expression compared with the CBL1 expression level in the wild-type (Ws) plants under normal conditions.

Using these complemented lines, we investigated whether the overexpression of SjCBL1 resulted in recovering the salt tolerance. To this end, we germinated the seeds of wild-type (Ws), *cbl1* mutant, and complemented (*cbl1/SjCBL1*) plants on MS agar medium and then transferred 4-day-old seedlings to MS agar plates supplemented with or without 125 mM NaCl for vertical growth. As expected, the *cbl1* mutant seedlings displayed retarded root growth and severe leaf chlorosis under the high salt condition as compared with the wild-type plants (**Figure 10B**). However, such salt-sensitive phenotypes were not observed in the seedlings of the complemented plants. In fact, the complemented (*cbl1/SjCBL1*) plants grew better than the *cbl1* mutant seedlings on the high salt medium, showing better root growth with more branches.

We also extended the salt stress assay to their adult plants by applying 150 mM NaCl solution to 3-week-old plants every 3 days. As illustrated in **Figure 10C**, the stem of the *cbl1* plants showed a drastic growth arrest and eventually collapsed 12 days after the treatment. Furthermore, the rosette leaves of the mutant plants turned yellow as quantitatively demonstrated by their chlorophyll content (**Figure 10D**). In contrast, *cbl1/SjCBL1* plants retained normal stem elongation and produced many siliques filled with numerous seeds. As a matter of fact, the complemented plants generated more seeds (∼122 mg/plant) than Ws (∼103 mg/plant) under the salt stress condition. However, both plants produced essentially the same amount of seeds (∼146 mg/plant) with no discernable differences under non-stressful growth conditions.

**FIGURE 11:**
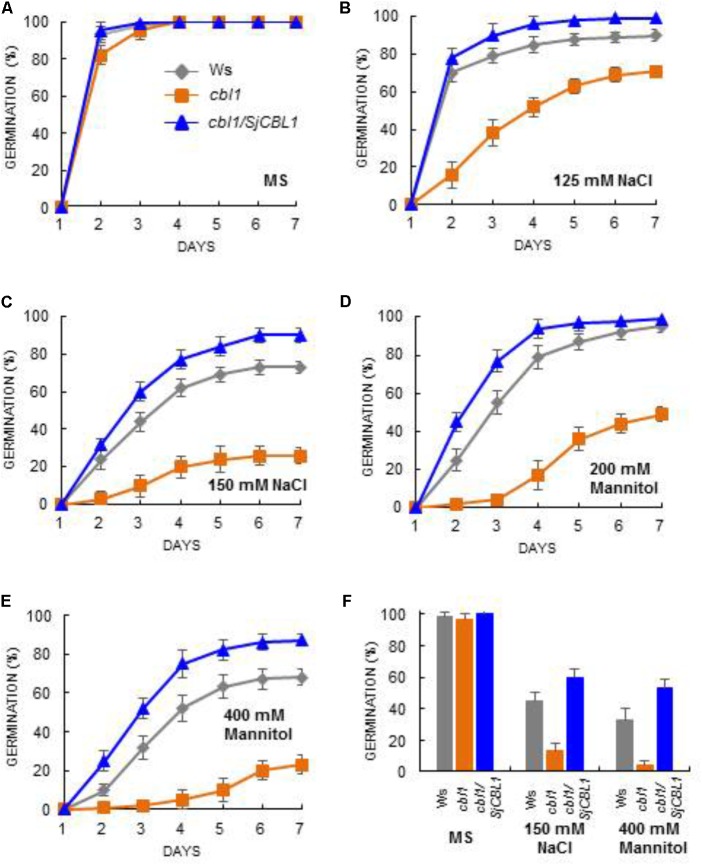
Seed germination analysis. Arabidopsis seeds of wild-type (Ws), *cbl1* mutant, and *cbl1*/SjCBL1 complementation plants were plated on MS agar medium **(A)** and MS medium supplemented with 125 mM NaCl **(B)**, 150 mM NaCl **(C)**, 200 mM Mannitol **(D)**, or 400 mM Mannitol **(E)**. The plates were incubated at 4°C for 3 days before transfer to 23°C for germination. Germination time course (days of incubation at 23°C) was recorded for Ws (rhombuses), *cbl1* (squares), and *cbl1*/SjCBL1 plant (triangles) seeds. **(F)** Seed germination rates in Ws, *cbl1*, and *cbl1/SjCBL1* on MS medium (control) or MS medium containing 150 mM NaCl or 400 mM mannitol. Germination was scored at 3 days after incubation at 23°C. Results are presented as average values ± SE from three independent experiments.

To further corroborate this observation, we carried out seed germination assays by dispersing the seeds of Ws, *cbl1*, and *cbl1/SjCBL1* plants on MS medium containing two different concentrations of NaCl and mannitol as described in **Figure 11**. On the normal medium (MS), their germination rates were not significantly disparate from each other (**Figure 11A**). On the stress media, however, they exhibited distinct germination patterns in a 7-day period (**Figures 11B–E**): Apparently, Ws and *cbl1/SjCBL1* seeds had germinated at much higher rates than *cbl1* mutant seeds. In addition, it is also clear that *cbl1/SjCBL1* seeds had retained significantly higher germination rates than Ws seeds on MS media supplemented with 150 mM NaCl or 400 mM mannitol. As summarized in **Figure 11F**, on 150 mM NaCl medium, ∼60% of *cbl1/SjCBL1* seeds had germinated in 3 days but only ∼40% of Ws seeds had germinated. In the case of 400 mM mannitol, *cbl1/SjCBL1* and Ws seeds displayed about 50 and 30% germination rates, respectively. It should be noted that *cbl1* seeds had germinated less than 10% in both conditions.

Taken together, these results from the complementation analyses indicated that ectopic expression of SjCBL1 could fully rescue the salt and osmotic stress-sensitive phenotype of Arabidopsis *cbl1* mutant plants, which is comparable to the Arabidopsis *cbl1/AtCBL1* complementation lines (Cheong et al., 2003). Therefore, it is obvious that SjCBL1 can act as a functional ortholog of AtCBL1 in *Sedirea japonica*.

## Discussion

Salinity and drought are major environmental stresses that limit the growth and development of economically important crops and flowering plants worldwide. Therefore, it is important to isolate and characterize genes that mediate the stress response in these plants, which can eventually lead to the development of salt- and drought-tolerant plants. In this regard, research findings obtained from the Arabidopsis model plants provide useful information to begin with. For example, AtCBL1 is involved in mediating Ca^2+^ signals elicited by a variety of abiotic stresses, and overexpression of AtCBL1 increases tolerance to salt and drought in transgenic Arabidopsis plants (Albrecht et al., 2003; Cheong et al., 2003). For an efficient translational biology approach on this useful gene, we have developed in this study a novel way to isolate AtCBL1-like genes from a variety of plant species without any genomic sequence information.

We compared available CBL1 cDNA sequences from different plants and discovered a highly conserved region. Based on this finding, we designed two moderately degenerate primers (MDP1 and MDP2) which can specifically anneal to the evolutionarily conserved sequences of the CBL1 cDNAs. Using these two primers, we performed 3′ RACE followed by 5′RACE and successfully isolated the 1.0-kb CBL1 cDNA from *Sedirea japonica* (*SjCBL1*), an orchid plant species whose genome sequence is currently unknown. Sequence analysis (**Figure 2B**) revealed that the SjCBL1 cDNA clone indeed encodes a polypeptide very similar to AtCBL1 in several aspects: SjCBL1 and AtCBL1 consist of 214 amino acid residues sharing 92% similarity. In addition, both proteins also harbor the *N*-myristoylation motif (Batistic et al., 2008), four EF-hand Ca^2+^-binding motifs (Kawasaki et al., 1998; Akaboshi et al., 2008), and the C-terminus PFPF motif (Du et al., 2011).

It is interesting to note that the most diverse region between SjCBL1 and AtCBL1 lies in the N-terminal end as shown in **Figure 2B**. Such evolutionary differentiation can provide an explanation why SjCBL1 and AtCBL1 exhibited different patterns of interaction with the Arabidopsis 26 CIPK family members (**Figure 4B**). In fact, our previous work demonstrated the importance of the N-terminal region of CBL members in determining the interaction affinity (Kim et al., 2000): Sequence comparison of 10 CBL members in Arabidopsis revealed that their N-terminal region is most variable and responsible for conferring the interaction specificity toward the CIPK family members.

We empirically demonstrated that SjCBL1 is a functional ortholog of AtCBL1 in the orchid plants by conducting a series of experiments: First of all, the yeast two-hybrid assays (**Figure 4**) showed that SjCBL1 can interact with the Arabidopsis CIPK family members such as AtCIPK1, AtCIPK23, and AtCIPK24, which are well-known interactors of AtCBL1 (Shi et al., 1999; Kolukisaoglu et al., 2004; D′Angelo et al., 2006; Li et al., 2006; Xu et al., 2006). Second, the gel-shift and pull-down assays (**Figure 7**) indicated that the recombinant SjCBL1 protein actually possesses the Ca^2+^-binding activity *in vitro* and can forms a complex with AtCIPK1 in a Ca^2+^-dependent manner as demonstrated in AtCBL1 (Kudla et al., 1999; Shi et al., 1999). Third, like AtCBL1, the SjCBL1 protein carrying the N-myristoylation motif was localized at the plasma membrane of plant cells (**Figure 6**), and there it physically interacted with AtCIPK1 (**Figure 8**). Finally, the stress-sensitive phenotype of the Arabidopsis *cbl1* mutant plants was fully rescued by the ectopic expression of SjCBL1 (**Figures 10**, **11**).

Among 10 members of the CBL family in Arabidopsis, AtCBL1 is most similar to AtCBL9. It is well known that both calcium sensors can activate the CIPK23 enzyme activity in a calcium-dependent manner, thereby phosphorylating AKT1 (K^+^ transporter) to enhance K^+^ uptake under low- K^+^ soil conditions (Li et al., 2006; Xu et al., 2006). Due to the close relatedness, AtCBL1 and AtCBL9 seem to be redundant in activating CIPK23 to mediate low K^+^ response: Unlike the CIPK23 knock-out Arabidopsis mutant (*cipk23*) plants hypersensitive to low- K^+^ concentrations, loss-of-function mutations in either CBL1 (*cbl1*) or CBL9 (*cbl9*) failed to render such sensitive phenotype. However, the *cbl1/cbl9* double mutant plants displayed the hypersensitive phenotype similar to *cipk23* (Li et al., 2006; Xu et al., 2006).

In spite of this functional redundancy, AtCBL1 and AtCBL9 appear to have distinct functions as well. Although AtCBL1 is not implicated in ABA signaling, AtCBL9 plays an important role in both the biosynthesis and sensitivity of ABA in Arabidopsis (Albrecht et al., 2003; Cheong et al., 2003; Pandey et al., 2004). This difference can provide a molecular mechanism by which AtCIPK1 mediate ABA-independent and ABA-dependent stress responses by forming an alternative complex with either AtCBL1 or AtCBL9 (D′Angelo et al., 2006). Therefore, isolation and characterization of the SjCBL9 and SjCIPK1 genes will be an important task to determine whether Arabidopsis CBL1/CBL9-CIPK1 pathway is conserved and acts similarly in *Sedirea japonica*.

In the present study, we opened the door for elucidating the CBL-type calcium sensor mediated signaling pathways in *Sedirea japonica.* Our findings strongly suggest that the emerging CBL-CIPK Ca^2+^-signaling networks should exist in the orchid plant and play critical roles in responding to a variety of environmental stresses as unraveled in Arabidopsis (Kim, 2013; Yu et al., 2014). Certainly, future studies need to demonstrate the existence of CIPK family members in *Sedirea japonica* (SjCIPKs) in order to verify conclusively such signaling mechanisms. In this regard, it is noteworthy that SjCBL1 displayed high interaction affinities toward the three AtCIPK members, AtCIPK1, AtCIPK23, and AtCIPK24, which are respectively involved in mediating osmotic, low K^+^, and high salt stresses in Arabidopsis (D′Angelo et al., 2006; Li et al., 2006; Xu et al., 2006). Using SjCBL1 as a stepping stone, it would be relatively easy to isolate the SjCIPK member genes despite the fact that no genomic sequence is available for *Sedirea japonica*. In fact, SjCBL1 can serve as bait in a yeast two-hybrid screen to identify cDNA clones that encode SjCBL1-interacting partner proteins such as the SjCIPK family members.

In summary, we have developed two degenerate primers, MDP1 and MDP2, which can be used as universal primers for amplifying the CBL1 cDNA from a number of plant species without the aid of the genomic sequences. As a matter of fact, we successfully cloned the CBL1 gene in *Sedirea japonica* (SjCBL1) using these primers. Therefore, this work greatly facilitates cloning of the CBL1 genes in many other plants that do not have available genomic sequences. Furthermore, it will significantly contribute to broadening and deepening our understanding of the CBL1-mediated Ca^2+^ signaling networks in the plant kingdom, which can be utilized to produce useful crop and horticultural plants with enhanced tolerance to abiotic stresses such as high salt and drought.

## Author Contributions

K-NK conceived and designed the research. JC, MC, and KY conducted the experiments. K-NK and JC analyzed the data. K-NK wrote the manuscript with contributions from JC. All the authors read and approved the final manuscript.

## Conflict of Interest Statement

The authors declare that the research was conducted in the absence of any commercial or financial relationships that could be construed as a potential conflict of interest.

## Abbreviations

BiFC: bimolecular fluorescence complementation
CaMV: cauliflower mosaic virus
CBLs: calcineurin B-like proteins
CIPKs: CBL-interacting protein kinases
GST: glutathione S-transferase
RACE: rapid amplification of cDNA ends
RT-PCR: reverse transcription polymerase chain reaction
YFP: yellow fluorescence protein
